# A plan for systematic reviews for high-need areas in forensic science

**DOI:** 10.1016/j.fsisyn.2024.100542

**Published:** 2024-08-31

**Authors:** Jason M. Chin, Anna-Maria Arabia, Merryn McKinnon, Matthew J. Page, Rachel A. Searston

**Affiliations:** a College of Law, Australian National University, Australia; b Australian Academy of Science, Australia; c Centre for the Public Awareness of Science, Australian National University, Australia; d School of Public Health, Monash University, Australia; e School of Psychology, The University of Adelaide, Australia

## Abstract

Forensic scientific practitioners and researchers must navigate a rapidly growing body of research. This makes it increasingly challenging to inform courts, lawyers, and other decision makers about the state of the field, thus heightening the chances of wrongful convictions and acquittals. When similar challenges have arisen in other fields, they have turned to systematic reviews, which are research reviews that use formal, articulated methods to provide a comprehensive summary of the literature on a specific research question. Systematic reviews allow users to evaluate how the authors identified research and synthesised its findings, making them more transparent than informal literature reviews. This article lays out a justification and plan for systematic reviews in forensic science.

The essence of [expert evidence] is that it draws on accumulated sources of information and the product of research of others recorded in professional publications [1]

## 1 Introduction

As the above quote from a recent Australian appellate court decision expresses, expert evidence depends on the research that founds it [1]. This is not particular to Australia, but is based on the general structure of expert knowledge [2]. That is, the expert's ultimate opinion is often just the tip of an iceberg built on many unstated decisions about what research to rely on and what research to disregard. In many fields, forensic science included, research bases are rapidly increasing [3,4]. This new knowledge is essential to the healthy functioning of the legal system, but it also presents challenges. Expert witnesses are required to summarise the research their opinion is based on. Yet, like all of us, their time and resources are limited. So, when research is being rapidly produced and published, it increases the burden on those experts. Moreover, as more research is produced, it becomes increasingly difficult to avoid citing that research in a way that confirms one's preferred view. This commentary proposes an evidence-based remedy to ease these mounting pressures on forensic scientists.

When cognate public-facing fields have faced similar challenges, they have turned to systematic reviews [5]. Systematic reviews are research reviews that use formal, transparently reported methods for identifying relevant research and synthesising its findings. In other words, systematic review authors document the methods used to identify studies, select studies for inclusion, collect study data, and analyse results. Accordingly, these reviews can be updated by the original review team or by others as new research is conducted, producing efficiencies. Systematic reviews are also verifiable in that users can see how research was found and selected for inclusion (i.e., they are reproducible). This increases the public accountability of those producing the systematic review [6].

Accordingly, systematic reviews may assist with the increasingly difficult job that forensic scientists face in staying on top of research and summarising it for legal decision makers. In making this case, we will start by giving a brief background into the duties of expert witnesses because we see systematic reviews as helpful in fulfilling these duties. We then further describe systematic reviews and discuss a plan for how interdisciplinary teams of forensic scientists and research synthesists may produce them. We end with reflections on how these systematic reviews can benefit many actors in the legal system.

Note that the plan we lay out in this essay complements other initiatives and perspectives discussed in this special issue about communicating forensic science. For example, systematic reviews can play an important part in Ballantyne and colleagues' annexures for expert reports [7]. They also assist in communicating error rates, as discussed by Martire and colleagues in this issue [8]. Finally, systematic reviews offer answers to Heavey and Houck's [9] concerns about the credibility of forensic science. That is, rigorous systematic reviews can assist forensic science in both aligning its practices with other scientific fields and better fulfilling the needs of the “wider justice organism” [9].

## 2 The legal context: empirical frameworks and their applications

The legal system expects that expert witnesses will assist the court by providing knowledge that will help in making a factual determination [10]. This includes performing a diverse array of tasks that are regularly the subject of expert evidence: comparing two fingerprints and determining whether they appear to have come from the same source [11], explaining the factors that can affect an eyewitness's memory [12], and determining whether a wound was self-inflicted or not [10]. Looking at these tasks, we can see that they can be separated into two parts [13]. First, the expert provides evidence of the “framework” they are relying on [13]. This is the “accumulated sources of information and the product of research of others” mentioned in the opening epigraph [1]. Then – in some cases – the expert applies that general framework to the case at hand.

In the example of the fingerprint examiner, the framework part of their evidence includes information about the process of comparing two fingerprints, validation studies demonstrating the accuracy of that process, and any threats to that process, such as cognitive bias [7]. This evidence is largely a summary of research [2]. Then, they explain how they applied that framework to the instant case by following that research-supported procedure. In other cases, experts are *only* permitted to provide the framework, leaving it to the judge or jury to determine if and how it applies. This includes psychologists who provide framework evidence about situational factors that affect the reliability of eyewitness identifications generally, but do not apply that knowledge to the case [12,14]. It also includes experts who opine on the typical mental state of drug traffickers, as in the recent U.S. Supreme Court case, *Diaz v US* [14].

In all cases, however, experts must “furnish the Judge or jury with the necessary scientific criteria for testing the accuracy of their conclusions, so as to enable the Judge or jury to form their own independent judgment by the application of these criteria to the facts proved in evidence” [10,15]. In some jurisdictions, where judges must gatekeep (i.e., exclude) evidence when it is not demonstrably reliable, these scientific criteria help judges make that decision about whether evidence is reliable enough to go to the factfinder [16]. In jurisdictions where the evidence rules are more hands off, it is still essential that experts provide the knowledge needed to test the accuracy of their conclusions. This is because the factfinder cannot rationally assign weight to the expert's opinion without such background.

A rigorous and clearly expressed research summary is essential to furnishing the judge and jury with the scientific criteria needed to rationally evaluate expert evidence. This includes drawing the court's attention to research that would cast doubt on their opinion, such as research finding a forensic practice is vulnerable to cognitive bias [7]. References to research that provide this context and caution are required both by the evidence law principles quoted above and by expert witness codes of conduct in many jurisdictions [7,17]. However, as the following section details, these requirements are increasingly difficult to fulfil because of the proliferating amount of research in many fields, including forensic science.

## 3 The growing “research culture” in forensic science

Forensic science is developing a “research culture”, which has been defined as a:

culture in which the question of **the relationship between research-based knowledge and laboratory practices is both foregrounded and central**. We mean a culture in which the following questions are primary: What do we know? How do we know that? How sure are we about that? We mean a culture in which these **questions are answered by reference to data, to published studies, and to publicly accessible materials**, rather than primarily by reference to experience or craft knowledge, or simply assumed to be true because they have long been assumed to be true. [18, **emphasis added**]

Much of this culture change was driven by the discovery of many wrongful convictions based on forensic scientific practices and a critical National Academy of Sciences (NAS) report [19] calling for, among other things, more research.

These calls have, to some degree, been successful. That is, some in the forensic science community have begun testing their practices and publishing subsequent reports in peer-reviewed journals [20].

Conducting and publishing new research helps satisfy some of the legal system's needs. As noted above, published research is at the heart of the empirical frameworks that are the foundation of most expert evidence. However, one unintended result of this flow of new research is that it has become a “daunting task to keep track of the relevant literature” [4]. Wading through this research places a strain on both forensic science researchers and practitioners (and those wearing both hats, see Ref. [21]). Researchers, who are charged with building a cumulative research base, must do more work to identify what research has been conducted and where the gaps are.

Practitioners, especially those providing evidence for legal proceedings, are also affected. They have the increasingly difficult task of providing framework evidence; they must summarise what is known and unknown about their expanding field in a way that is understandable to the factfinder. One example of the difficulties facing practitioners can be found in the New South Wales case, *JP v DPP* [11]. In that case, the witness, a fingerprint examiner, noted that they only had time to review the research that was forwarded to them by their employer, the New South Wales Police:

**Defence Counsel**: Do you keep abreast of the current available scientific research in relation to fingerprint identification?

**Fingerprint Examiner**: I read available documentation that I have at Dubbo Crime Scene when time permits.

**Defence Counsel**: Do you stay up to date with the science related to fingerprint examination identification?

**Fingerprint Examiner**: I read documents that are sent to me by the training area periodically.

**Defence Counsel**: Is it your evidence that in relation to your expertise and staying up to date with the fingerprint field you basically rely on whatever the New South Wales Police training section sends to you?

**Fingerprint Examiner**: Updated versions and methodology yes.

**Defence Counsel**: You don't do anything of your own initiative to remain up to date in the field, is that right?

**Fingerprint Examiner**: Unfortunately time does not permit within my area [22].

Given the difficulties raised by a proliferating research base, it may be no surprise that there are emerging efforts aimed at addressing them. Notably, Ballantyne and colleagues [7] report in this issue on Victoria Police's project to include “annexures” with all of the forensic reports it provides. These annexures include summaries of the research underlying the forensic practice or an acknowledgement that there is little or no existing research. As Ballantyne and colleagues note, this includes: “the findings of the [National Academy of Sciences] and the President's Council of Advisors on Science and Technology (PCAST) reports where relevant to the discipline and includes information about any progress made since these reports, including relevant validation or error rate studies” [7]. Moreover, they note that their annexures will be updated “regularly […] as additional knowledge is gained through research and publications” [7].

As can be seen, Victoria Police's annexures rely heavily on research summaries conducted by learned academies and governmental bodies. The aforementioned NAS report, for instance, reviewed several fields of forensic science and found a lack of research. Seven years later, the PCAST conducted a similar review, finding that some progress had been made towards conducting research on widely used practices. It then synthesised that research. Besides serving as the basis for some of Victoria Police's annexures, these reports have provided important knowledge to courts and, as noted, inspired the current research culture within forensic science [18,20,23,24].

Unfortunately, those large reports are not being regularly updated and they are not easy to update. This is because they were not conducted in a transparent and reproducible way – the authors did not report how they searched the literature to find the research and gaps in research they identified, nor did they report how decisions were made as to what to include or exclude in the review. There are good reasons for this in that those reports were designed with different purposes in mind than those of systematic reviews (see below under “The role of learned societies”). In any event, the result is that Victoria Police (and any organisation wishing to follow suit) faces a challenging task. The research summaries they initially relied on are out of date and impossible to replicate, leaving them with the difficult task of keeping track of the research underlying its 52 annexures.

Moreover, some in the forensic science community have rejected the findings of reports from oversight bodies. For example, the Association of Firearm and Tool Mark Examiners (“AFTE”) rejected the finding of the PCAST Report, saying “[…] we cannot overstate our disappointment in the PCAST's choice to ignore the research that has been conducted” [25,26]. While it is difficult to avoid allegations of bias, transparency of methods may assist. The next section suggests that the transparent and verifiable nature of systematic reviews are a way to manage the challenges we have just outlined.

## 4 Systematic reviews

To deal more effectively with its growing research base, forensic science should look to how other fields have managed this challenge. Here, research from the fields of medicine and education provide an apt example [5]. These fields are similar in that they regularly inform public decision making (e.g., by helping doctors determine what drugs to prescribe) and they have also grappled with growing research bases. To manage these forces, researchers began producing what are known as “systematic reviews”.

In short, a systematic review is a review that uses formal, explicitly stated methods to collate and synthesise findings of studies that address a clearly formulated question [27]. Authors of systematic reviews report how the literature was searched, ideally presenting the full Boolean search strings used. They also report the databases that were searched. The review authors then explain what eligibility criteria were used to determine whether to include a study and how results were extracted and synthesised (e.g., using meta-analysis). As a result, the review's results are both verifiable by outsiders and reproducible because the methods can be reused in the future to see if the results change as new research is produced.

Consider, for example, the question of whether anti-inflammatory drugs can contribute to dementia prevention. Medical professionals may wonder whether there is sufficient evidence to recommend that their patients take anti-inflammatories [28]. Rather than rely on the results of one study, they might turn to an informal literature review of various studies to more fully evaluate the existing evidence. However, an informal review finding no studies or only a few studies showing weak evidence would not be very useful to that doctor because they could not be confident that the review authors searched sufficiently to identify the relevant research evidence. That is, an absence of studies in an informal review is not very diagnostic on its own – we need to know whether or not the search had the ability to identify relevant studies. Moreover, the doctor might be concerned about the possibility of conflicts of interest among the review authors. Perhaps, for example, several of the review authors had received funding from pharmaceutical companies. The doctor might reasonably worry that these review authors would be inclined to exclude studies that show the drugs are ineffective.

Systematic reviews are extremely useful in situations like these. In this example, there is indeed a systematic review studying the effect of anti-inflammatories on dementia prevention, finding no benefit [28]. However, the doctor would not be forced to simply take the review authors' word for it. Rather, they would be able to confirm that the review authors searched databases likely to house research on dementia and that they used search terms that would find such research. Moreover, they could also check to see if the review authors changed important aspects of their process, such as their inclusion or exclusion criteria midway through their review, possibly in reaction to what they were finding. They could do this by checking the systematic review's “registration”, which is a public, timestamped protocol for the review. As a result, the doctor can be more confident that any conflicts of interest held by the review author did not affect their review.

Since the development of systematic reviews, their methodologies have been subject to continuous refinement by researchers who specialise in systematic review methodology. For instance, in 2009, a group of methodologists, clinicians, and journal editors developed the PRISMA (Preferred Reporting Items for Systematic reviews and Meta-Analyses) Statement, a set of best practices for reporting systematic reviews [29]. It was updated in 2020 with additional practices and tools for reporting systematic reviews [27]. More generally, research synthesis has developed into a field in its own right: “University departments, international bodies such as the Cochrane Collaboration (a leading producer of systematic reviews on health topics), and numerous conferences and journals have established scientific methods, conventions and production systems for evidence synthesis” [30].

There is also growing evidence that expertise in conducting formal reviews of research provides a demonstrable benefit to the process. For instance, studies of systematic reviews in medicine [31], dentistry [32], and education [33] find that including a research synthesis specialist or librarian in the process is associated with several benefits. This research finds, for example, that systematic reviews with authorship teams that include review experts better report their methodology (i.e., comply better with PRISMA) [31] and are more likely to search beyond standard databases [34]. Not surprisingly then, Cochrane recommends that “Review teams should also include expertise in systematic review methodology” [35]. The Campbell Collaboration, which produces systematic reviews on social science topics, provides similar guidance [36].

These studies also underscore the fact that not all reviews that purport to be “systematic” live up to that label. That is, many systematic reviews, even those that claim to fully comply with PRISMA, fail to report basic aspects of their search and synthesis process [37]. The problem has become significant enough that researchers produced a “living” systematic review (i.e., a systematic review that is updated over time as new research is collected) that currently contains 485 articles documenting issues found with published systematic reviews [37]. Improving the reporting and quality of systematic reviews is important because – as we have mentioned – users often place more weight on systematic reviews.

**Fig. 1:**
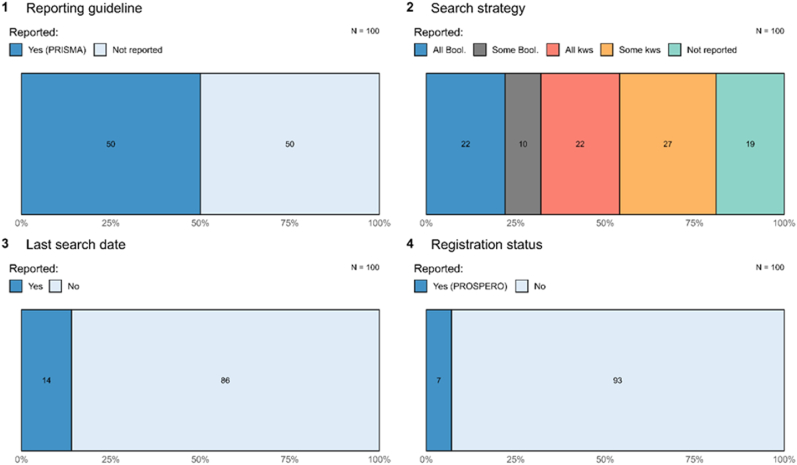
The transparency of forensic science systematic reviews **Figure caption**. Data, code, and registration information needed to reproduce this figure are available at https://osf.io/9v8un/.

Within forensic science specifically, there is reason to think that many purportedly ‘systematic’ reviews are not actually transparent or reproducible [38]. For instance, a review of 100 systematic reviews published in forensic science from 2018 to 2021 revealed considerable unevenness in the actual reporting of these reviews (despite all claiming to be systematic) (see Fig. 1) [38]. For instance, half reported following a reporting guideline (PRISMA), but claiming to follow this reporting guideline was only modestly related to actually following it. As Fig. 1 shows, only 22 followed best practices in reporting all of their Boolean search strings, 14 reported their last search date, and 7 reported being registered.

Limited uptake of transparent and reproducible reporting in forensic science systematic reviews is worrisome for many reasons. Many users might assume that these “systematic” reviews use methods that can be verified by others and place unwarranted trust in them. Other users – such as courts – might be frustrated because they would want to know how recently searches were run in order to know if they are relying on current or out-of-date information. And, other forensic scientists might want to update (i.e., reproduce) the results of these reviews over time, but would not be able to because the methods were never clearly articulated. This contributes to considerable waste.

## 5 A plan for systematic reviews in forensic science

There appears to be a serious need for systematic reviews in forensic science to respond to its growing research culture.^1^ Reports drafted by oversight bodies have focused on reporting consensus documents rather than using systematic review methods. And, purportedly systematic reviews being published in forensic science journals do not seem to be following best practices such that they are transparent and reproducible [38]. This section lays out a way to fill in the gap. In short, it proposes a plan for interdisciplinary synthesis teams to produce fully transparent systematic reviews in high need areas of forensic science (we discuss what might be considered high need below). It also envisions the creation of workflows and reproducible methods for future systematic reviews. These reviews can help improve research by identifying gaps and can help practitioners, such as Victoria Police, by providing reliable research summaries for their expert reports and annexures.

First, Cochrane and other organisations make clear that producing high quality systematic reviews requires expertise. To address this, forensic scientists should collaborate with research synthesists. As we saw, teams that include such expertise tend to produce more transparent, reproducible, and thorough systematic reviews. Forensic scientists seeking synthesis collaborators may find such individuals in Cochrane or in the evidence synthesis units that sit in many universities.

Next, forensic scientists should consider the *what*, the areas that are most in need of a high quality systematic review. There are many factors that might be considered here. For instance, they might focus on areas that have seen a great deal of research over the past several years, that is, those that are especially “daunting” [4] to stay on top of. Systematic reviews in this area can help in many ways. They can assist in getting a sense of what is currently known and they will be crucial in identifying gaps in the research that need filling in.

On the other hand, some systematic reviews might be guided by the needs of other stakeholders [39]. For instance, if a relatively new forensic practice begins appearing in court, it may be sensible to conduct a systematic review to determine the strength of its evidence base. Additionally, synthesist teams may choose to focus on practices that frequently appear in court. Recall that while many feature comparison practices have been informally reviewed, the review methods were never made available [19,20]. As a result, they cannot be reproduced as more research is produced. Producing a rigorous, reproducible systematic review for fingerprint analysis, for example, could be useful because it could be updated as new knowledge is produced about this widely used technique and its limits (e.g., studies measuring how accuracy is affected by the quality of the latent print).

Regarding *how* systematic reviews in forensic science ought to be conducted and reported, emerging forensic science synthesis teams should engage in as much transparency as is feasible. To be maximally useful (and efficient to update), these reviews ought to follow research synthesis's highest standards for transparency and reproducibility. This includes following a reporting guideline, such as PRISMA. In the long run, however, forensic science may wish to develop a consensus reporting guideline of its own. It may include guidance specific to forensic science, such as the importance of contacting large laboratories for datasets [40] and norms specific to evaluating validation studies in forensic science [20].

The review process for systematic reviews in forensic science should also be as transparent as possible. Review authors should follow the registered report model used by Cochrane and others whereby the systematic review protocol is peer reviewed and registered prior to the review being executed. This will allow review authors to help improve the review before resources have already been expended [41]. It will also help ensure that any changes made to the search protocol are made apparent to users and justified. Open peer review (e.g., publication of the peer reviews along with the review) may also be useful so that users can readily see what critiques were made and how the interdisciplinary synthesis team responded.

Transparency of methods and open peer review may help promote the legitimacy of these systematic reviews. Recall that even large and seemingly rigorous reviews by government bodies have received limited buy-in among some forensic scientists and stakeholders, such as the Association of Firearm and Tool Mark Examiners [25,26]. While it is impossible to completely avoid allegations of biased selection of research, transparency may assist in assuaging some concerns. Stakeholders, such as the Association of Firearm and Tool Mark Examiners, will be able to assess the full synthesis process and observe that the synthesists held themselves to a high standard of transparency.

### 5.1 The role of learned societies

As noted, the U.S. National Academy of Science's 2009 review of forensic science was remarkably influential in inspiring change in forensic science. Relatedly, the U.K. Royal Society began its ongoing “Primers for Court” series in 2017 [42]. Entries in this series (e.g., “Forensic gait analysis: a primer for courts”) provide brief reviews of forensic science topics and issues for a legal audience.

Neither effort, however, was conducted as a systematic review, making them difficult to update. Accordingly, neither has been regularly updated. We do not point this out to suggest neglect on the part of the authorship teams. Rather, producing a systematic review did not align with these projects’ goals at the time. That is, the NAS convened a large body of stakeholders to attempt to produce a consensus document and the Royal Society is producing readily understandable guides that do not purport to cover the field.

Still, we note that learned societies are institutions that are well-placed to begin leading systematic reviews for forensic science. As we have seen, their existing efforts have proven useful to forensic science and the legal system. This may be because they are independent bodies not aligned with a particular party in the legal system, instead being primarily interested in advancing science in society. This is especially important in accusatorial systems, where we have seen predictable positions taken by groups aligned with the prosecution and defence [26]. Moreover, through their membership and convening power, learned academies have access to leading scientists.

By producing future reviews as systematic reviews, learned societies can avoid some of the pushback we saw against the PCAST Report [25,26]. That is, the Association of Firearm and Tool Mark Examiners and others seem to have viewed the PCAST Report as the *PCAST's consensus* rather than *the field's consensus*. Additional transparency may help avoid similar critiques. Indeed, this is the role that PRISMA plays for health research: “PRISMA helps to foster and further perpetuate the autonomy and public legitimization of biomedical research” [6]. For example, PRISMA's transparency guidelines include registering search and identification protocols prior to evaluating the results. This practice demonstrates to the community that the review authors are taking steps to avoid bias and promote scrutiny of their methods.

### 5.2 The role of cognitive scientists and science communicators: maximising comprehensibility of systematic reviews

High quality reviews in forensic science require systematic review expertise and transparency, but they are unlikely to be effective unless they are comprehensible to the justice system's diverse array of stakeholders. Indeed, there is ample evidence that people, including medical and legal professionals, frequently misunderstand the kind of statistical information found in systematic reviews and their abstracts [43]. For instance, advanced law students and judges in training are prone to misinterpret statistical information in DNA evidence [44]. When statistical information is presented in systematic review abstracts, which is often the case, these misunderstandings are especially troubling. This is because readers under time pressure, such as lawyers and forensic practitioners, may only read these high-level summaries and not scrutinise them further.

The need for improved communication of forensic science is not new, but rather has been highlighted as an area of concern for over a decade [45]. Although efforts have been made to improve the manner in which scientific evidence is presented, there is little systematic research about how to present summaries of scientific evidence to ensure understanding by judges, jurors and other stakeholders [46]. In fact, a review recently concluded that “right now there is very little evidence on the best way to present systematic review evidence to policymakers” [47]. We will now review several applicable findings and concepts from cognitive science and science communication that may apply, but that require further testing in the systematic review context.

One relevant concept is cognitive fluency, which refers to the ease with which readers can process information. More cognitively fluent content is associated with more positive affective judgments (e.g., perceptions of trust, likeability, credibility) [48] and greater comprehension [49]. Forensic expert opinion evidence is notoriously laden with scientific jargon [50]. Review authors can aid the fluency with which readers process key information in their reviews by limiting the use of scientific jargon and employing narrative techniques (e.g., analogy, concrete examples, visual aids) to communicate important details in a clear manner. Improving cognitive fluency is the “bread and butter” of science communication, which works to make complex scientific information relevant and understandable to a non-specialist audience. This can occur in a range of different contexts for a range of different purposes: to inform, entertain, and/or influence behaviours and decision-making [51].

Relatedly, cognitive load refers to the amount of information that a reader can process at one time. Cognitive load theory suggests that learning or comprehension is hampered when cognitive demand exceeds the reader's capacity to process information [52]. Review authors can help to prevent cognitive overload and enhance reader retention of crucial information by minimising unnecessary information, trading comprehensiveness for comprehensibility.

Failing to communicate scientific evidence properly can contribute to a loss of trust in scientific evidence. Non-specialist audiences need to trust specialists to provide reliable information to help inform decisions about the most appropriate actions to take [53]. Cues to trust studied by scientific communication specialists include disclosing uncertainties and providing information about the quality of the evidence presented [[54], [55], [56]]. For example, science communication studies have shown that communicating uncertainty can increase perceptions of trustworthiness of information sources [56].

Here, we can see that systematic review authors will have to carefully balance communicating uncertainty with communicating information in a fluent and concise way (to avoid cognitive overload). To do so, systematic review authors for forensic science can build on international movements that are developing more effective structural approaches to communicating expert opinion, including their uncertainties [46].

Another concept relevant to how review summaries may be consumed and used is the negativity bias, which relates to the emotional processing of content. Negative information is more heavily weighted than neutral or positive information [57] and, as a result, the emotional valence (positive vs. negative) of word choices can have a significant impact on comprehension, memory, and even propensity to share information [58]. For instance, science abstracts with more negative words than positive are better understood and remembered [59]. Review authors may leverage the negativity bias by using negative words at key parts of review summaries to capture readers’ attention and create a more accurate lasting impression of key details.

These concepts are a small sampling of insights from cognitive science and science communication that review authors could borrow to inform their communication of review findings. Note, however, that there remains a gap in research testing how best to present summaries of science to stakeholders in the justice system for optimal comprehension and decision-making. Future research applying cognitive science and science communication concepts to this problem will help to ensure that summaries of scientific results are both comprehensible and actionable for decision-makers.

## 6 The wider benefits of systematic reviews for the justice system

We have mostly discussed the benefits of systematic reviews for forensic scientific researchers and practitioners. For researchers, this included systematic reviews helping them to stay on top of the literature and to allocate research funds more efficiently. For practitioners, systematic reviews can assist in the important task of providing reliable information to decision makers. However, systematic reviews can also support the work of other actors in the legal system.

First, accused people and their lawyers will benefit from high quality systematic reviews. Defence lawyers in many jurisdictions are typically under-resourced, especially as compared to the prosecution [[60], [61], [62]]. This “adversarial deficit” [62] often means that they rely on limited legal aid funds that do not provide for retaining a counter-expert. Such experts can be helpful in several ways, such as in providing a fuller view of the research behind a forensic practice and assisting the defence lawyer with their litigation strategy (e.g., planning their approach for cross-examining the prosecution's forensic expert). This deficit is becoming increasingly impactful as more research is published for at least two reasons: there is more research for the prosecution expert to selectively refer to and there is more research for the defence lawyer to try to understand.

While systematic reviews are no substitute for a human expert, they can ease adversarial deficit's harmful impacts. For instance, rigorous systematic reviews will help defence lawyers prepare for their cross-examination, ensuring they are not surprised by research they were not aware of [63]. Systematic reviews may also have a prophylactic effect. Prosecution experts are less likely to selectively refer to research if they are aware that all parties are well informed of the relevant literature.

Systematic reviews also assist prosecutors and investigators. Prosecutors have a duty to lead relevant evidence [64]. This can be challenging with expert evidence because it may not be obvious to lay prosecutors that there is research (or an absence of research) that is exculpatory and should be brought to the court's attention. Indeed, prosecutors have been susceptible to allegations of misconduct in such cases [64]. Rigorous yet comprehensible systematic reviews help avoid this situation by providing a quick guide to what is known and unknown about a practice. Similarly, investigators have a considerably better chance of identifying the correct perpetrator (and not wasting time following false leads) when they can calibrate their investigation to the strength of the available evidence.

Judges are required to maintain the fairness of trials [65,66] and gatekeep expert evidence when it is unreliable [67]. All of the forces outlined above (e.g., adversarial deficit, poorly calibrated investigations) put pressure on judges to safeguard trials when other mechanisms have failed. In these situations, a systematic review can provide a trustworthy starting point for determining if a forensic practice is sufficiently reliable to assist the factfinder.

One final stakeholder worth mentioning is the public itself. It is in the public's interest to see legal decisions informed by the most up-to-date and reliable science so that the correct person can be prosecuted and a wrongful conviction or acquittal avoided. If decisions are based on flawed and outdated information, then the public may lose faith in the justice system.

## 7 Conclusion

Expert knowledge is essential to the healthy functioning of the justice system. But when knowledge builds rapidly, difficult questions arise for forensic practitioners regarding how to find, identify, and organise that knowledge in a way that judges and juries can digest. Similar challenges arise for researchers seeking to build a cumulative science. In fields such as health and education, systematic reviews have emerged as an important tool for transparently representing what is known and unknown about a topic. In this article, we have suggested and supported the notion that systematic reviews can play a similar role in both forensic science and in the legal system that it serves. That is, we laid out a plan for interdisciplinary teams of research synthesists, forensic scientists, and learned societies to come together to produce high quality systematic reviews of key forensic practices. Such reviews can both further accelerate knowledge generation and help forensic scientists fulfil their duty to fairly assist the court.

## Funding disclosure

Searston: ARC Industry Fellowship (IE230100380)

## CRediT authorship contribution statement

**Jason M. Chin:** Writing – review & editing, Writing – original draft, Project administration, Conceptualization. **Anna-Maria Arabia:** Writing – review & editing, Writing – original draft, Conceptualization. **Merryn McKinnon:** Writing – review & editing, Writing – original draft, Conceptualization. **Matthew J. Page:** Writing – review & editing, Writing – original draft, Conceptualization. **Rachel A. Searston:** Writing – review & editing, Writing – original draft, Conceptualization.

## Competing interest disclosure

The authors declare the following financial interests/personal relationships which may be considered as potential competing interests:

**Jason M. Chin**: **While** I declare no competing financial or personal relationship, I am the Registered Reports for this journal. Accordingly, an independent editor handled the peer review of this article.

## Footnotes

1 To clarify, we are referring to the definition of systematic reviews used above, those that use formal, articulated methods [27,29] to review research. Informal reviews, such as those that are regularly published as part of the *Annual Reviews* series have value, but are not our focus.
